# Early “reduction to practice” of the CRISPR–Cas9 invention in eukaryotic cells

**DOI:** 10.3389/fgene.2022.1009688

**Published:** 2022-10-04

**Authors:** Guillermo Aquino-Jarquin

**Affiliations:** Laboratorio de Investigación en Genómica, Genética y Bioinformática, Torre de Hemato-Oncología, 4to Piso, Sección 2, Hospital Infantil de México, Federico Gómez, Ciudad de México, Mexico

**Keywords:** CRISPR-Cas9 applications, inventive step, reduction to practice, substancial evidence, Broad Institute, CVC

## Introduction

The CRISPR (Clustered Regularly Interspaced Short Palindromic Repeats)–Cas (CRISPR-associated proteins) are microbial adaptive immune mechanisms that have revolutionized many areas of Life Sciences research and innovation and potentially could transform the lives of patients with limited medical treatment options. Although CRISPR’s seminal contributions range from fundamental explorations to the first demonstrations of CRISPR**–**mediated genome editing in eukaryotic cells, current discoveries using CRISPR likely represent “the tip of the iceberg.”

Several CRISPR–Cas systems require multiple proteins to function. Notably, the type II systems found in many bacteria require a single endonuclease known as Cas9, a CRISPR RNA (crRNA), and a *trans*-activating CRISPR RNA (tracrRNA), which form a functional DNA-targeting complex (Mojica et al., 2009). Charpentier & Doudna simplified the crRNA and the tracrRNA by combining these transcripts into a single molecule known as a single-guide RNA (the sgRNA). They were the first to show that sgRNA is sufficient for programming Cas9 to direct the nuclease activity to any target site (Jinek et al., 2012).

Molecular Biologists have quickly adopted this bacterial immune system into eukaryotic systems to modify the genome of practically any organism with unprecedented ease. In principle, through the CRISPR–Cas9 systems, double-strand breaks (DSB) can be induced in any given chromosomal region-of-interest (Jinek et al., 2012; Kleinstiver et al., 2015), followed by repair of the target site *via* Non-Homologous End Joining (ligation of DNA-ends with potential incorporation of insertions and deletions into the sequence) or Homology-Directed Repair (the exchange of genetic information between DNA segments with similar sequences) mechanisms (Doudna and Charpentier, 2014). This allows a range of permanent modifications, including eliminating entire genes or pathogenic DNA variants and inserting therapeutic genes; thus, CRISPR could treat or even cure severe genetic disorders (Porteus, 2019).

## A controversial battle on the intellectual property rights to the CRISPR–Cas9 technology

**FIGURE 1:**
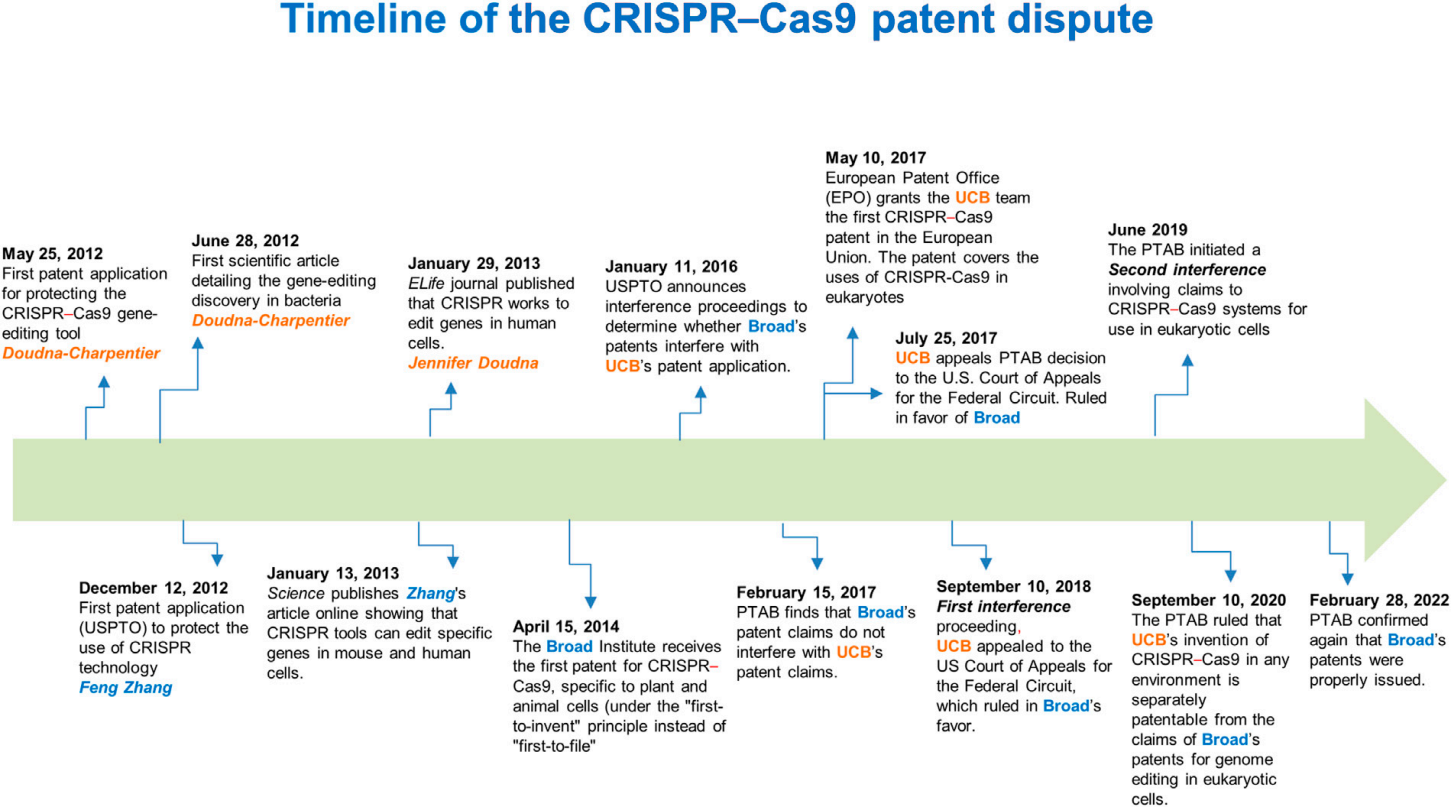
Timeline of the CRISPR–Cas9 patent dispute. A 10-year timeline of fundamental scientific contributions and patent decisions on the first CRISPR–Cas9 inventions. USPTO, U.S. Patent and Trademark Offic*e*; PTAB, Patent Trial and Appeal Board; EPO, European Patent Office; UCB, University of California, Berkeley.

There has been a 7-year long dispute between “Broad”^1^ (Feng Zhang) and “CVC”^2^ (Jennifer Doudna–Emmanuelle Charpentier) about who possesses exclusive patent rights for the foundational CRISPR–Cas9 genome-editing technology in eukaryotic cells. When Broad received its first CRISPR patent on 15 April 2014 (Figure 1), CVC claimed that this was patent interference with the same invention, in that Doudna-Charpentier filed the first patent application on 25 May 2012, which led to a legal dispute. An “interference proceeding” is a lawful procedure exclusive to U.S. patent law that attempts to ascertain whether two related patents claim the same invention. If so, such a process determines which party was the first to invent it (Sherkow, 2017).

On 28 February 2022, after two interference proceedings and other appeals (Figure 1), the Patent Trial and Appeal Board (PTAB) and U.S. federal courts issued a judgment and decision (Decision on Priority), establishing the claims that Broad’s patents for genome editing methods in eukaryotic cells are patentably distinct. The latter implies that their results are not reasonably expected from CVC’s *in vitro* and in bacterial systems experiments (Sherkow, 2022). Concerning this, although Doudna and Charpentier first published evidence that the CRISPR system could be used as an RNA-programmable genome editing tool (28 June 2012), 7 months before the Broad team led by Feng Zhang did, Doudna and Charpentier did not show in the initial paper that the system worked in eukaryotic cells (Jinek et al., 2012). In this regard, the CVC’s invention claims the design of the RNA molecule that guides the critical step in CRISPR–Cas9 gene editing, directing the Cas9 nuclease to a specific site in the genome. Nevertheless, achieving the system’s functioning in eukaryotes was an additional inventive step that ultimately awarded the court ruling to the Broad Institute (Broad Communications, 2022; Sherkow, 2022).

Is Broad’s work in mammalian cells “obvious” in light of Doudna’s work in bacterial systems, or did it possess an “inventive step” to render it separately patentable? According to Jacob Sherkow, professor of law at the University of Illinois, Urbana-Champaign, the review in the CRISPR patent dispute was the “substantial evidence” standard (Sherkow, 2018). This means that a fact-finder examiner based its decision on substantially sufficient evidence for this to be reasonable (Sherkow, 2018).

The question then arises of why CVC could not provide this “substantial evidence” at the time. Interestingly, details of experiments conducted by Doudna and Charpentier (as specified on pages from dated lab notebooks) revealed difficulties in inducing their invention to work, suggesting that the eukaryotic experiment would not succeed (Sherkow, 2022). In this regard, based on the PTAB decision, a “person skilled in the art” (e.g., the average Molecular Biologist) would not have reasonably expected the CRISPR–Cas9 system to succeed in a eukaryotic environment without “substantial evidence” of its functionality (Sherkow, 2022).

In analyzing the context of this “substantial evidence” that was crucial to the decision made by the attorneys, we would have to focus on critical aspects for the CRISPR–Cas9 system to work in a eukaryotic environment. In this respect, as Cas9 is a bacterial protein, suitable codon usage was required to create a “humanized” Cas9 version (altering its encoded sequence, but without changing the amino-acid composition and protein structure), therefore, enhancing Cas9 activity in eukaryotic cells, including human cells. Furthermore, redirecting the Cas9 protein from the cytoplasm into the nucleus of eukaryotic cells to carry out editing events also required incorporating a short functional peptide known as nuclear localization signal (NLS) without affecting the structure and function of Cas9. Feng Zhang’s group codon-optimized the Cas9 nuclease from *Streptococcus pyogenes* (*Sp*Cas9) and attached an NLS to ensure nuclear compartmentalization in mammalian cells (Cong et al., 2013). Thus, the Feng Zhang group reconstituted the non-coding RNA components of the *S. pyogenes* type II CRISPR–Cas system by the incorporation of expression vectors (known as plasmids) for producing an 89-nucleotide tracrRNA under the RNA polymerase III U6 promoter (a regulatory region commonly used to express small RNAs in eukaryotic cells) (Cong et al., 2013). Thus, this strategy carried out efficient RNA-guided genome modification in mammalian cells (Cong et al., 2013). Accordingly, these improvements in the CRISPR–Cas system employed by Zhang’s group were not “obvious” extensions of the work of Doudna & Charpentier, whose scope was limited to cutting purified DNA in cell-free environments (Jinek et al., 2012), based on the patent application filed. Therefore, such evidence led the examiners to conclude that Broad’s claims and those of the CVC’ do not interfere with each other and that the inventive step claimed by Broad is not derived from experiments conducted in test tubes, stated in the first and second interference proceedings (Sherkow, 2022) (Figure 1).

Such technical hurdles (“experimental uncertainty”) that the CVC presented in getting their invention to work (the so-called “reduction to practice” of the invention) probably gave Broad the upper hand in showing that this CRISPR–Cas9 system worked in human and mouse cells (Cong et al., 2013), regardless of whether the CVC conceived the idea first.

Finally, the evidence obtained by Jennifer Doudna’s group, confirming that the CRISPR–Cas9 system works to edit genes in eukaryotic cells, was published online in *eLife* on 29 January 2013 (Jinek et al., 2013). Nevertheless, Feng Zhang’s group had first published such a demonstration [manuscript submitted to *Science* on 5 October 2012 and published online on 3 January 2013 (Cong et al., 2013); (Figure 1)]; thus, Broad maintains priority in its demonstration of use.

Interestingly, the other two papers describing the application of CRISPR–Cas9 gene-editing technology in eukaryotes had already been published in January 2013 (Hwang et al., 2013; Mali et al., 2013) prior to the filing of the patent application (December 2013) by Broad at the European Patent Office (EPO). However, according to the EPO, Broad’s patent claims were no longer entirely novel because the technology was already in the public domain (Harrison, 2018). Therefore, Broad’s patent application was discarded in the European Union. By contrast, CVC has the upper hand in Europe, which has issued patents concerning the CRISPR–Cas9 systems in over 30 countries, unaffected by any U.S. interference proceedings (Sanders, 2022).

In case of a new appeal by CVC (possibly by a third interference), it is unlikely to persuade the attorneys and claim sole ownership of the CRISPR patent if CVC does not provide “substantial evidence” demonstrating the application of the first-time CRISPR system in eukaryotic cells (and if the decision of the court is based only on this).

Based on Broad Institute statements (Broad Communications, 2017), Broad Institute could grant licenses for using CRISPR non-exclusively and through the open ‘inclusive innovation’ model for therapeutic development across many human diseases (where this technology can be applied) instead of obtaining commercial licenses (Broad Communications, 2017). This model offers one license for its exclusive use for 2 years. After this period, there is an open call for applications by Parties seeking to license the CRISPR–Cas9 technology for application through the Broad website (Broad Communications, 2017). Furthermore, Broad has encouraged establishing a worldwide CRISPR–Cas9 licensing pool or a coordinated licensing approach, such as the joint licensing framework (an agreement that has made CRISPR–Cas9 technology available non-exclusively) previously developed for the use of CRISPR in agriculture (Broad Communications, 2022).

Based on the UC Berkeley website (Sanders, 2022), CVC has more than 40 issued U.S. patents that were not implicated in this Decision on Priority, which involve various applications of CRISPR–Cas9 genome editing systems in different environments, including eukaryotic cells (Sanders, 2022). However, biotech start-ups such as *CRISPR Therapeutics* (co-founded by Charpentier), *Caribou Biosciences*, and *Intellia Therapeutics* (co-founded by Doudna) might require obtaining licenses with Broad to apply this technology in therapeutic interventions for further research and development plans, probably through the open “inclusive innovation” model.

## Concluding remarks

The discovery of new CRISPR–Cas systems (orthologs or Cas9 equivalents) or the engineering of new optimized systems for genome editing will lead to application for new patents. In those cases, the following critical questions arise: To what extent could the functions of new CRISPR–Cas systems be considered "non-obvious" regarding the original inventions of CRISPR–Cas9? Because experimental uncertainty is more in line with the realities of scientific research [based on Doudna’s hurdles to achieving “reduction to practice” of the CRISPR–Cas system in eukaryotic cells (Sherkow, 2017)], should patent-law legal and regulatory framework consider the “unpredictability of success” for future CRISPR patents and not only issue a pragmatic judgment (based on “substantial evidence”)?

The panorama is not entirely clear. The first demonstration of genome editing ability by the CRISPR–Cas9 system in a cellular environment different from the bacterial one could be considered substantially more “unpredictable” (Sherkow, 2017). This implies that the sgRNA interaction with Cas9 nuclease and the consequent recognition of the target site to be edited in a eukaryotic system involves several variables that require assessment in terms of the performance and reproducibility of the experiments. In this regard, Molecular Biology tends to be substantially more “unpredictable” (even when there are excellent theoretical arguments that the system can work properly), which could lead to the outcome of any experiment conducted being uncertain (Sherkow, 2017). In this scenario, achieving the functioning of a bacterial system in a eukaryotic system could take time due to technical or methodological difficulties, even though Molecular Biologists have great expertise in performing the experiments. Due to the latter, it is difficult to establish whether a “person of ordinary skill in the art” would interpret the invention as “obvious” or as lacking an “inventive step” (Sherkow, 2017), which represents a relevant aspect that should be considered in issuing rulings.

However, Molecular Biologists have learned from the first descriptions and characterizations of CRISPR–Cas systems. In the case of new nucleases for genome editing applications, it is feasible to implement different laboratory techniques and novel experimental strategies to investigate whether CRISPR–Cas systems function correctly in eukaryotic systems (despite their bacterial origin) and under other conditions in which these CRISPR–Cas systems are tested for the first time. After discovering new Cas effector proteins, their biochemical characterization is faster, and their functionality is rigorously evaluated in *in-vitro* and *in-vivo* models. Thus, the therapeutic breakthrough of these CRISPR tools might be rapidly incorporated into new gene-editing clinical studies, where perhaps the therapeutic success rate of this technology can be estimated.

However, although many CRISPR-based treatments in advanced clinical phases would be available in the short term, beyond new litigation, CVC and Broad should resolve the exclusivity issue for human therapeutics and ensure that such CRISPR-based therapies maximize patient benefit, considering significant ethical, safety, and societal aspects of this technology.

## Footnotes

1 “Broad” (Broad Institute/Harvard University/the Massachusetts Institute of Technology).

2 “CVC” (the University of California Berkeley/the University of Vienna/Emmanuelle Charpentier).
